# Cost-Effectiveness Analysis of Test-Based versus Presumptive Treatment of Uncomplicated Malaria in Children under Five Years in an Area of High Transmission in Central Ghana

**DOI:** 10.1371/journal.pone.0164055

**Published:** 2016-10-03

**Authors:** Theresa Tawiah, Kristian Schultz Hansen, Frank Baiden, Jane Bruce, Mathilda Tivura, Rupert Delimini, Seeba Amengo-Etego, Daniel Chandramohan, Seth Owusu-Agyei, Jayne Webster

**Affiliations:** 1 Kintampo Health Research Centre, Kintampo, Brong Ahafo Region, Ghana; 2 Department of Global Health and Development, London School of Hygiene and Tropical Medicine, London, United Kingdom; 3 Section of Health Services Research, Department of Public Health, University of Copenhagen, Copenhagen, Denmark; 4 Epidemiology Unit, Ensign College of Public Health, Kpong, Eastern Region, Ghana; 5 Department for Disease Control, London School of Hygiene and Tropical Medicine, London, United Kingdom; Centro de Pesquisas Rene Rachou, BRAZIL

## Abstract

**Background:**

The presumptive approach of confirming malaria in health facilities leads to over-diagnosis of malaria, over use of anti-malaria drugs and the risk of drug resistance development. WHO recommends parasitological confirmation before treatment with artemisinin-based combination therapy (ACT) in all suspected malaria patients. The use of malaria rapid diagnostic tests (mRDTs) would make it possible for prescribers to diagnose malaria at point-of-care and better target the use of antimalarials. Therefore, a cost-effectiveness analysis was performed on the introduction of mRDTs for management of malaria in under-five children in a high transmission area in Ghana where presumptive diagnosis was the norm in public health centres.

**Methods:**

A cluster-randomised controlled trial where thirty-two health centres were randomised into test-based diagnosis of malaria using mRDTs (intervention) or clinical judgement (control) was used to measure the effect of mRDTs on appropriate treatment: ‘a child with a positive reference diagnosis prescribed a course of ACT or a child with a negative reference diagnosis not given an ACT’. Cost data was collected from five purposively selected health centres and used to estimate the health sector costs of performing an mRDT and treat children for malaria and other common febrile illnesses. Costs of training healthcare personnel and supervision in the study period were also collected. A sample of caregivers to children participating in the trial was interviewed about household cost incurred on transport, drugs, fees, and special food during a period of one week after the health centre visit as well as days unable to work. A decision model approach was used to calculate the incremental cost-effectiveness ratios (ICERs). Univariate and multivariate sensitivity analyses were applied to assess the robustness of ICERs.

**Results:**

The availability of mRDTs for malaria diagnosis resulted in fewer ACT treatments compared to the clinical judgement approach (73% versus 81%) and more children appropriately treated (70% versus 57%). The introduction of mRDT-based diagnosis would cost the Ministry of Health US$18.6 per extra appropriately treated child under five compared to clinical judgement while the ICER from a societal perspective was lower at US$11.0 per appropriately treated child. ICERs were sensitive to a decrease in adherence to negative mRDTs, malaria positivity rate and specificity of the mRDT.

**Conclusion:**

The introduction of mRDTs is likely to be considered cost-effective in this high transmission setting as this intervention increased the number of appropriately treated children at low cost.

**Trial Registration:**

ClinicalTrials.gov NCT00832754

## Background

Malaria remains a major cause of morbidity and mortality in sub-Saharan African countries with an estimated 188 million cases and 395,000 deaths in 2014 of which the majority occurred in children below 5 years [1]. The use of the laboratory tests to confirm cases of malaria has been a challenge for many years and the levels of transmission justified the presumptive approach to its management [2]. However, presumptive management led to the over-diagnosis of malaria and over use of antimalarial drugs in many settings and consequently contributed to the selection of parasites resistant to chloroquine and sulphadoxine-pyrimethamine [3–6]. Over-diagnosis of malaria also led to the delayed appropriate treatment of non-malarial febrile illnesses (NMFI) [7–11]. The World Health Organization (WHO) recently updated the guidelines on malaria case management to recommend parasitological confirmation before treatment in all suspected malaria patients including children under five years of age [12].

The use of malaria rapid diagnostic tests (mRDTs) would make it possible for prescribers to diagnose malaria at point-of-care and better target the use of antimalarials. This approach is only now being scaled up in Ghana and the presumptive use of antimalarials in the management of malaria is still practiced in many primary care facilities in the country. While the introduction of mRDT diagnosis in a setting with presumptive diagnosis of malaria will likely improve the targeting of antimalarials to children with parasites, the added cost of the mRDTs is also an important consideration.

Although microscopy performed by a competent laboratory technician under ideal conditions is the gold standard for the diagnosis of malaria [13], its accuracy under operational conditions in Africa can be quite low [14]. Several studies have shown that mRDT diagnosis is more cost-effective than presumptive diagnosis in an era of expensive antimalarials such as artemisinin-based combination therapy (ACT) [13,15–21]. This finding is however sensitive to several factors of which malaria transmission level and adherence to negative test results by healthcare providers appear as the most important [15,19,20,22].

A recently concluded cluster-randomised controlled trial in a high transmission region of central Ghana evaluated the effect of restricting ACT treatment to mRDT-positive cases in under-five children seeking care in primary public health centres [23]. The objectives of the trial were to estimate the effect of mRDT introduction on appropriate treatment of malaria and on the incidence of malaria or anaemia among under-five children. It was concluded that test-based management of malaria improved quality of care, increased the number of children appropriately treated and did not increase the risk of malaria among children in the area compared to presumptive diagnosis of malaria. The present research built on this trial to develop a cost-effectiveness analysis assessing the impact of introducing mRDTs for the treatment of under-five children in a high transmission setting where presumptive diagnosis was the norm in public health centres. This research will contribute to knowledge on the cost-effectiveness of introducing mRDTs in a high transmission setting since previous cost-effectiveness analyses have typically been conducted in low to medium transmission areas [13,16,19,21]. The trial utilised for the cost-effectiveness analysis was registered in the online clinical trials registry as NCT00832754 (ClinicalTrials.gov).

## Methods

### Study area

A detailed description of the study area has been published previously [24]. Briefly, the study was conducted in Kintampo North Municipality, Kintampo South district, Techiman Municipality, Nkoranza North and South and Tain districts in the Brong Ahafo region of Ghana. It involved 32 health centres in the five districts. The catchment populations of these health centres range from 10,000 to 85,000. Malaria transmission in these areas is high and occurs all-year round, and malaria is the leading diagnosis among under-fives in outpatient departments in public health centres [25,26].

### Study design

The study was a cost-effectiveness analysis which used data from a cluster-randomised, controlled trial which compared the effects of mRDT-based diagnosis (intervention) versus clinical judgement based-diagnosis (control) for treatment of malaria in public health centres in rural Ghana [23]. In the intervention arm, children were offered ACT only when the mRDT result was positive while in the control arm, children were given ACT on the basis of presenting clinical signs and symptoms. In health centres of both arms, the approach to child assessment, classification and treatment for non-malaria illnesses followed guidelines of the Integrated Management of Childhood Illness (IMCI).

In order to avoid the possible effect of financial barrier, the research project paid and ensured that all the children participating in the trial were enrolled under the Ghana National Health Insurance Scheme.

As recommended in the economic literature [27], the societal perspective was chosen for the cost-effectiveness analysis and therefore included the costs to all those involved in the management of febrile illness including the health sector and the households of children suffering fever episodes. A cost-effectiveness analysis from a health sector perspective was also performed to enable District Health Management Teams and country level health policy makers to appreciate the estimated costs (and effects) of introducing mRDTs to the health system at the health centre level.

### Training

Health personnel attending to sick children at health centres in both arms participated in workshops where training in the management of malaria and other febrile illnesses according to IMCI guidelines were offered as well as training on how to perform mRDTs (intervention arm only). This involved both didactic presentation of the guidelines and discussion sessions on how to implement them. Training was conducted before the start of trial and repeated every 4–6 months until the end of the study. District field supervisors paid regular visits to participating health centres. Whenever a new health worker was transferred into any of the facilities, the research team were alerted by the stationed fieldworker and training was organized and conducted.

At each of the health centres, a trained research field officer was the first person to meet a member of the cohort who was subsequently guided through the processes at the facility leading to attending by the healthcare personnel in the outpatient department. Quarterly review meetings were held with the healthcare personnel and the research field officers at the health centres. During these meetings, the objectives of the research, study allocation and the importance of adherence were repeated to the participants and refresher training was also provided.

### Sampling

The unit of randomisation for the study arms was public health centres. Thirty-two health centres were stratified by districts, ranked within districts by the number of DPT1 vaccinations given in the facility in the preceding year and paired according to the rank. From each pair of health facilities one was randomly allocated to the intervention or control arm. All households located within a 2 km radius of study health centres were enumerated and one hundred children below 24 months of age living closest to the health centres were enrolled. Household cost data was collected from children enrolled in 10 of the 32 health facilities (5 intervention and 5 control). Health facility cost data was collected from 5 purposively selected health centres based upon logistical feasibility and a range of sizes, where size was defined as out-patient attendance in 2010.

### Measurement of effect

The measure of effect was ‘appropriate treatment of malaria with ACT’ which was a composite indicator defined as a child with a positive reference diagnosis prescribed a course of ACT or a child with a negative reference diagnosis not given an ACT. Reference diagnosis was by blood slide double-read by two microscopists at the reference laboratory at the Kintampo Health Research Centre and with discrepancies resolved through a reading by a third microscopist.

Over the two-year period of the trial, 5573 fever episodes were reported to a health centre by children in the intervention arm while 5791 fever episodes by those in the control arm [23]. Information on malaria diagnosis by mRDT or clinical signs and symptoms as determined by health centre staff, together with the medicines prescribed were captured from all these visits. Fieldworkers took blood samples for smear microscopy for later reference diagnosis from each visit which enabled the classification of the treatment given as appropriate for malaria or not according to the definition specified above.

### Measurement of costs

#### Training and supervision costs

Costs of training workshops to provide healthcare personnel in the participating health centres with refresher training in the management of febrile illnesses and training on how to perform an mRDT (intervention arm only) were obtained from the study financial accounts that had been maintained continuously during the study period. Cost of supervision of health personnel at participating health centres was estimated by interviewing supervisors about the frequency of supervisory visits, time per visit and their salary level. Supervisory costs were estimated by study arm for the two-year trial period.

#### Health centre costs

Costs per service for a diagnosis by mRDT and treatment for malaria and common non-malarial febrile illnesses were estimated based on data collection in five public health centres in the study area. At each health centre, total recurrent expenditure for the financial year 2011 were obtained on salaries, drugs, disposables, stationery, maintenance of buildings, communication, utilities, transport and domestic expenses. This information was available either from health centres themselves or at the District Office. Capital costs in the financial year were estimated based on the construction costs of a standard health centre and 2011 prices of equipment and furniture found at the health centres. Since capital goods were used for a longer time than one year, annual equivalents of the total costs of these were calculated using expected lifespans of 30, 7 and 10 years for buildings, equipment and furniture respectively and a discount rate of 0.05 [27,28].

Total health centre costs for the financial year 2011 were allocated to final patient services using the standard step-down costing methodology [27,29]. Health centre costs by category were distributed to overhead cost centres (administration, accounting, cleaning) and intermediate cost centres (dispensary, mRDT storage, diagnostics) and finally to patient cost centres (direct patient care) in a step-wise fashion using allocation criteria expected to represent actual resource use. For instance, salary expenditure was allocated to cost centres according to where individual personnel spent their time on a typical day as captured through interviews.

Micro-costing methods [30] were used to supplement the step-down costing in order to calculate more accurately the services relevant for this study including the diagnosis of malaria using mRDT and treatment of malaria and non-malarial febrile illnesses in the outpatient department. The personnel responsible for performing mRDTs were interviewed on the time spent per test based on which the salary cost per mRDT diagnosis could be calculated. The price of the mRDT purchased for the study was US$0.82 as obtained from the study accounting system. Malaria and non-malarial febrile illnesses were treated in the outpatient departments of the health centres. Medical assistants and nurses were asked to estimate the required average time used for an outpatient visit to treat these illnesses including taking illness history, measuring temperature and prescribing drugs. Costs of drugs prescribed by diagnosis were estimated based on the assumption that healthcare workers generally followed official clinical guidelines and with cost per treatment dose obtained from Regional Medical Stores. Taking care not to double count, cost data from the step-down costing methodology and the micro-costing were combined to form full unit costs per mRDT test and outpatient treatments of malaria and non-malarial febrile illnesses in all five health centres visited.

#### Household costs

Household cost data was collected from September 2010 to June 2012 in a randomly selected sample of study children experiencing a fever episode. Main caregivers of the children were interviewed at the time of their visit to a study health centre with a febrile child and then again interviewed in their homes on days 3 and 7 by a trained interviewer. A total of 2006 febrile episodes was included for household cost interviews– 885 in the test-based arm and 1121 in the clinical judgement arm. The purpose of these interviews was to capture information on household costs for transport, drugs, consultation fees, laboratory fees and special foods purchased to improve the health of the child, incurred at the initial visit at the health centre, and additional treatment-seeking visits to any health provider to manage the febrile episode. To avoid double counting, any fees paid by the household to public health centres were excluded as resources for these services had already been captured in the health centre costing component. In addition, main caregivers were asked how much time had been used per trip for travelling to health providers and if the illness of the child had resulted in days where the main caregiver was unable to perform usual activities. This opportunity cost of time was valued at US$1.14 per person per day equal to the salary per day for females working in agriculture in rural areas in 2013 adjusted to the 2011 price level [31].

### Incremental cost-effectiveness analysis

**Fig 1:**
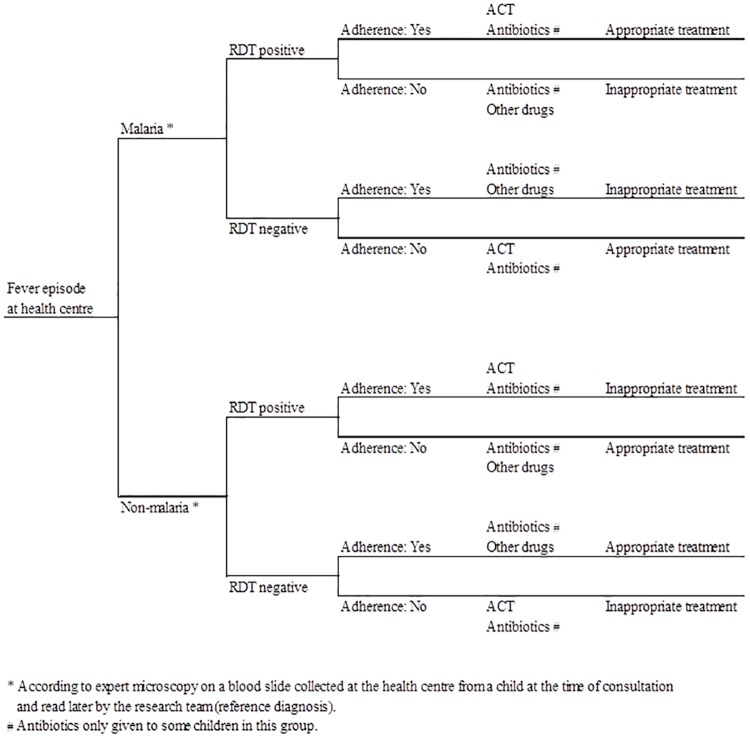
Decision tree for children with fever visiting public health centres offering malaria diagnosis by rapid diagnostic test, Kintampo District, Ghana.

**Fig 2:**
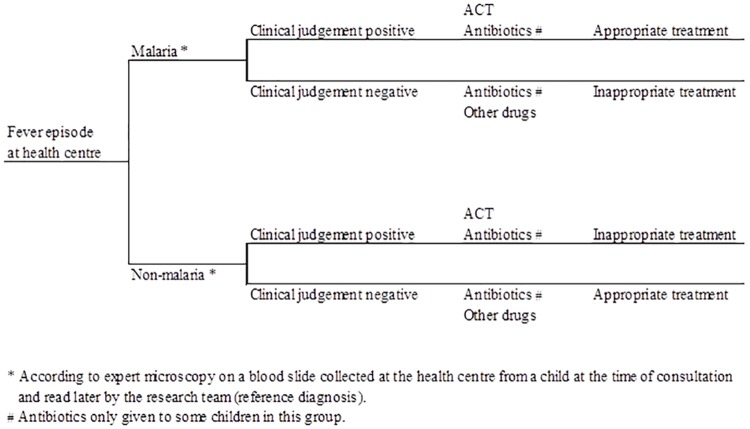
Decision tree for children with fever visiting public health centres offering malaria diagnosis by clinical judgement, Kintampo District, Ghana.

Costs were not collected at the individual level for all children enrolled in the trial [32] so the cost-effectiveness analysis was developed using a decision analytical approach [33]. Data on the costs and effects by arm were captured from a number of sources as described above and were linked utilising the decision trees by arm displayed in Figs 1 and 2. Probabilities of decision tree branches of malaria (reference diagnosis), mRDT or clinical diagnosis result and adherence to mRDT result were derived using outcomes from the cluster-randomised trial [23]. At the end of the decision tree, the treatment of fever episodes could be classified as appropriate or not according to the study definition (child with positive reference diagnosis prescribed a course of ACT or child with negative reference diagnosis not given an ACT). Some febrile children sought additional treatment after the first visit to a public health centre. The probability of seeking additional care within a one-week period was obtained from the household cost interviews. All probabilities used are listed in Table 1.

**Table 1:** Parameters utilised in decision model and distributions for probabilistic sensitivity analyses (PSA) for cost-effectiveness analysis of introducing malaria rapid diagnostic tests in public health centres in Kintampo District, Ghana, 2011 (US$1 = GHS1.51).

	------------ Value ------------			
Parameter	Test-based approach	Clinical judgement	Source	Distribution in PSA
Malaria prevalence among children with fever visiting health centres (%)	50.0	50.3	[23]	Point estimate
Sensitivity of diagnosis (%)	94.7	87.7	[23]	Beta
Specificity of diagnosis (%)	47.2	26.1	[23]	Beta
Adherence to positive mRDT result (%)	97.8	NA	[23]	Beta
Adherence to negative mRDT result (%)	96.3	NA	[23]	Beta
Training of health workers, cost per fever episode (US$)	1.7	1.6	λ	Point estimate
Supervision, cost per fever episode (US$)	0.4	0.4	λ	Point estimate
mRDT diagnosis in health centres, cost per visit (US$)	2.6	0.0	β	Point estimate
Treatment with ACT in health centres, cost per visit (US$)	5.9	5.9	β	Point estimate
Treatment with antibiotics in health centres, cost per visit (US$)	4.5	4.5	β	Point estimate
Treatment with other drugs in health centres, cost per visit (US$)	4.1	4.1	β	Point estimate
Probability of additional treatment seeking after health centre visit (%)	11.4	19.4	α	Beta
Out-of-pocket expenditure on additional treatment-seeking (US$)	2.0	1.8	α	Gamma
Opportunity cost of travel time for additional treatment-seeking (US$)	0.1	0.2	α	Gamma
Out-of-pocket expenditure on special foods, per fever episode (US$)	0.7	1.0	α	Gamma
Opportunity cost of days not able to work, per fever episode (US$)	4.6	5.1	α	Gamma
Value of lost time per day, assumption (US$)	1.1	1.1	[31]	Point estimate

^λ^ Study accounting system for the trial [23].

^β^ Primary data collection in five public health centres in Kintampo District. The mean of unit costs in five health centres.

^α^ Interview survey conducted among 2006 households in Kintampo District that experienced a fever episode in a child below 5 years and visited a public health centre.

Societal and health sector costs by study arm were calculated by attaching cost data to the decision trees with the cost data described above. Training and supervision costs estimated for the two-year trial period by study arm were divided by the number of fever episodes included during the trial time to arrive at an average unit cost per fever episode. Relevant services in public health centres included mRDT diagnosis and treatment in the outpatient department with ACT, antibiotics or other drugs. The mean of unit costs from five health centres were used in the decision models. Average household expenditure on travel, drugs and fees incurred during additional treatment-seeking within a one-week period after the initial visit to a public health centre were obtained from the household cost interviews in a sub-sample of trial participants. Time lost due to travelling to health providers, waiting and caring for ill children were self-reported from the household cost home interviews and valued at US$1.14 per day. All average costs per fever episode or health centre visit utilised in the decision models are listed in Table 1.

Total costs and effects were calculated for a simulated number of 1000 fever episodes in each study arm by letting these pass through the decision trees populated with event probabilities and cost data as described above. The incremental cost-effectiveness ratio (ICER) was obtained by subtracting total costs in the control arm from the total costs in the intervention arm (numerator) and subtracting the total effect measured as appropriately treated children in the control arm from total effect in the intervention arm (denominator). The ICER therefore showed the cost per additional appropriately treated fever episode when test-based management of malaria was introduced into public health centres which previously used clinical diagnosis.

### Sensitivity analysis

One-way sensitivity analyses were undertaken to examine how sensitive the ICER of introducing mRDT diagnosis in public health centres was to changes in important parameters and assumptions. These included malaria prevalence, mRDT accuracy, adherence to mRDT result by health workers, prices of mRDT and ACT, opportunity cost of lost time and a range of health sector unit costs. A two-way sensitivity analysis was performed on mRDT accuracy since an mRDT with high sensitivity will typically have a lower specificity and vice versa [34].

Probabilistic sensitivity analysis (PSA) was undertaken to assess the sensitivity of the ICER to simultaneous variation in relevant parameters [33]. This was done by defining relevant probability distributions to decision model parameters rather than point estimates. A beta distribution was assigned to all the branch probabilities used in the decision trees such as ‘adherence to mRDT result’ using trial data [23] to estimate distribution parameters. Gamma distributions were used to describe the variation in all household cost categories due to the non-negative, right-skewed nature of the data [33] and with distribution parameters estimated from the household cost survey interviews. The remaining cost parameters like health centre cost per visit entered the analysis as point estimates. An overview of all distributions and point estimates used in the PSA are listed in Table 1. Monte Carlo simulations were used to propagate uncertainty by simultaneously selecting values from each parameter distribution by running 8000 iterations in Excel (Microsoft, Seattle)–a number of iterations found to ensure reasonably stable mean ICERs [35]. Uncertainty surrounding the ICERs was summarised by plotting joint incremental cost and incremental effects in the cost-effectiveness plane and quantified by calculating confidence intervals and cost-effectiveness acceptability curves (CEACs) [36–39] where the latter showed the probability that the introduction of mRDTs in public health centres was cost-effective given different levels of a health policy maker’s hypothetical willingness to pay for an appropriately treated fever episode.

### Ethical issues

The study was approved by the ethics review committees of the Ghana Health Service, the Kintampo Health Research Centre and the London School of Hygiene and Tropical Medicine. Administrative approval was obtained from the respective district and hospital management teams, while health workers gave oral consent. Individual informed consent was obtained from all care givers. Signed (thumb printed if unable to read or write in English) informed consent was obtained from each caregiver prior to enrolment of the child. This consent procedure was approved by all mentioned ethical review committees.

## Results

The availability of mRDTs for malaria diagnosis resulted in fewer ACT treatments compared to the clinical judgement approach (73% versus 81%) and more children appropriately treated (70% versus 57%) according to the definition utilised in this study (Table 2). Health sector costs per 1000 fever episodes in the test-based arm were 31% higher than in the clinical judgement arm (US$10,484 versus $7,992) mainly due to the extra costs of the mRDTs and the extra personnel and other resources required to perform the test in public health centres. Cost savings from the lower proportion of febrile children treated with an ACT in the test-based arm compared to the clinical judgement arm was US$259 per 1000 fever episodes and covered only 10% of the extra cost of mRDT diagnosis.

**Table 2:** Cost and effects normalised to 1000 fever episodes by study arm and incremental cost-effectiveness ratio of replacing clinical diagnosis of malaria by rapid diagnostic tests in public health centres in Kintampo District, Ghana, 2011 (US$1 = GHS1.51).

	Test-based approach		Clinical judgement	
	***N***	%	***N***	%
*Fever episodes*	*1*,*000*	*100*	*1*,*000*	*100*
Treated with ACT	732	73	808	81
Appropriately treated ^#^	704	70	570	57
Reference diagnosis malaria positive	500	50	503	50
	***US$***	***%***	***US$***	***%***
*Health sector cost per 1000 fever episodes*	*10*,*484*	*64*	*7*,*992*	*54*
Training of health care workers	1,721	11	1,576	11
Supervision	478	3	590	4
mRDT diagnosis in health centres ^€^	2,616	16	0	0
Treatment with ACT in health centres ^β^	3,554	22	3,813	26
Treatment with antibiotics in health centres ^β^	1,944	12	1,865	13
Treatment with other drugs in health centres ^β^	171	1	147	1
*Household cost per 1000 fever episodes*	*5*,*873*	*36*	*6*,*891*	*46*
Drugs, fees, travel (out-of-pocket) ^λ^	395	2	580	4
Special foods (out-of-pocket)	700	4	1,049	7
Time lost (opportunity cost)	4,778	29	5,262	35
*Total societal cost per 1000 fever episodes*	*16*,*358*	*100*	*14*,*883*	*100*
**Incremental analysis (replace clinical judgement by test-based approach for 1000 fever episodes)**				
Incremental number of appropriately treated [95% CI]		134	[120; 148]	
Incremental health sector cost, US$ [95% CI]		2,492	[2,467; 2,517]	
Incremental societal cost, US$ [95% CI]		1,474	[-8,208; 11,767]	
ICER health sector perspective, US$ [95% CI]		18.6	[16.7; 20.9]	
ICER societal perspective, US$ [95% CI]		11.0	[-61.4; 87.3]	

^#^ Children with a positive reference diagnosis prescribed an ACT or children with a negative reference diagnosis not prescribed an ACT.

^€^ All resources required to perform diagnosis with mRDT in public health centre.

^β^ All resources required to conduct treatment in outpatient department in public health centre.

^λ^ First visit to a study health centre and subsequent treatment seeking to any health provider within 7 days.

With regards to costs borne by households, patients in the clinical judgment arm incurred considerably higher household cost per 1000 fever episodes than test-based arm (US$6,891 versus $5,873). This was due to the finding that a higher proportion of caregivers of children in the clinical judgement arm reported additional alternative treatment seeking (19% versus 11%, Table 1), more special foods were purchased and more days unable to do normal activities were experienced. The most important household cost component in both arms was the value of time lost by family caregivers for looking after their ill child. Total societal cost combining health sector and household cost for the test-based approach arm was US$16,358 for 1000 fever patients compared to US$14,883 in the clinical judgement arm. Household costs formed a high share of total societal costs in both arms; 36% in the test-based approach and 46% in the clinical judgement arm.

The introduction of diagnosis of malaria by mRDT instead of clinical judgement was estimated to lead to an increase of 134 appropriately treated patients (95% CI 120 to 148) in a population of 1000 fever patients (Table 2). The incremental cost of introducing mRDTs in public health centres per 1000 fever episodes was US$2,492 (95% CI US$2,467 to US$2,517) from a health sector perspective and US$1,474 (95% CI -US$8,208 to US$11,767) from a societal perspective. The ICER from a health sector perspective was found to be US$18.6 (95% CI US$16.7 to US$20.9) meaning that the introduction of mRDT-based diagnosis would cost the Ministry of Health US$18.6 per extra appropriately treated child under five compared to clinical judgement. The ICER from a societal perspective was lower at US$11.0 (95% CI -61.4 to 87.3) per appropriately treated child.

The one-way sensitivity analyses showed that the ICERs from both the health sector and the societal perspectives were robust to changes in the parameters investigated with a few exceptions (Table 3). A decrease in the adherence to negative mRDT results from the observed 96% to 70% or below increased the ICERs by more than 80% thereby making the introduction of mRDTs less cost-effective. Similarly, if the malaria prevalence among children with fever had been 80% rather than the observed 50%, then the ICERs would have been around 60% higher making mRDT diagnosis less attractive from a cost-effectiveness perspective. An improvement of mRDT specificity from 47% to 75% or above would decrease the ICERs by more than 50% thus resulting in mRDTs becoming more cost-effective relative to presumptive diagnosis. ICERs were less sensitive to the remaining individual parameters investigated. The two-way sensitivity analysis of mRDT accuracy varying simultaneously the sensitivity and specificity suggested that the ICERs were insensitive to this aspect (Table 3).

**Table 3:** Sensitivity to selected parameters of the incremental cost-effectiveness ratio (ICER) of replacing clinical diagnosis of malaria by rapid diagnostic tests in public health centres in Kintampo District, Ghana, July 2010-June 2012 (US$1 = GHS1.51).

	------- ICER in US$ -------			------- ICER in US$ -------	
Parameter ^&^	Health sector	Societal	Parameter ^&^	Health sector	Societal
*Malaria prevalence among children with fever (50%)*			*Health centre cost per visit excl drugs and mRDTs*		
20%	13.0	7.4	40% lower	13.9	6.3
30%	14.5	8.3	20% lower	16.2	8.7
40%	16.2	9.5	20% higher	20.9	13.3
60%	21.1	12.6	40% higher	23.3	15.7
70%	24.6	14.9	*Training and supervision cost per episode*		
*mRDT sensitivity (95%)*			40% lower	18,1	10.5
80%	36.3	20.7	20% lower	18.3	10.8
90%	21.9	12.8	20% higher	18.8	11.2
100%	16.0	9.6	40% higher	19.1	11.5
*mRDT specificity (47%)*			*Opportunity cost per day (US$1*.*14)*		
75%	8.6	4.8	US$0.00	18.6	14.6
90%	6.5	3.4	US$0.50	18.6	13.0
100%	5.5	2.8	US$1.00	18.6	11.4
*Sensitivity / specificity*			US$1.50	18,6	9.8
100% / 40%	20.7	12.5	US$2.00	18.6	8.2
90% / 50%	19.4	11.3	US$2.50	18.6	6.7
80% / 60%	18.2	10.0	*Probability of additional treatment-seeking in test-based arm (11%)*		
60%	46.5	28.7	5%	18.6	10.0
70%	33.5	20.4	20%	18.6	12.4
80%	25.9	15.6	30%	18.6	14.0
*mRDT price (US$0*.*82)*			*Probability of additional treatment-seeking in clinical judgement arm (19%)*		
US$0.20	13.6	6.0	5%	18.6	13.1
US$0.40	15.1	7.5	20%	18.6	10.9
US$0.60	16.6	9.0	30%	18.6	9.4
*ACT price (US$1*.*32)*			*Various household costs per episode*		
US$1.00	18.8	11.2	Special food equal by arm	18.6	13.6
US$1.20	18.7	11.1	Days lost equal by arm	18.6	14.3

^&^ Central parameter values are shown in parentheses.

**Fig 3:**
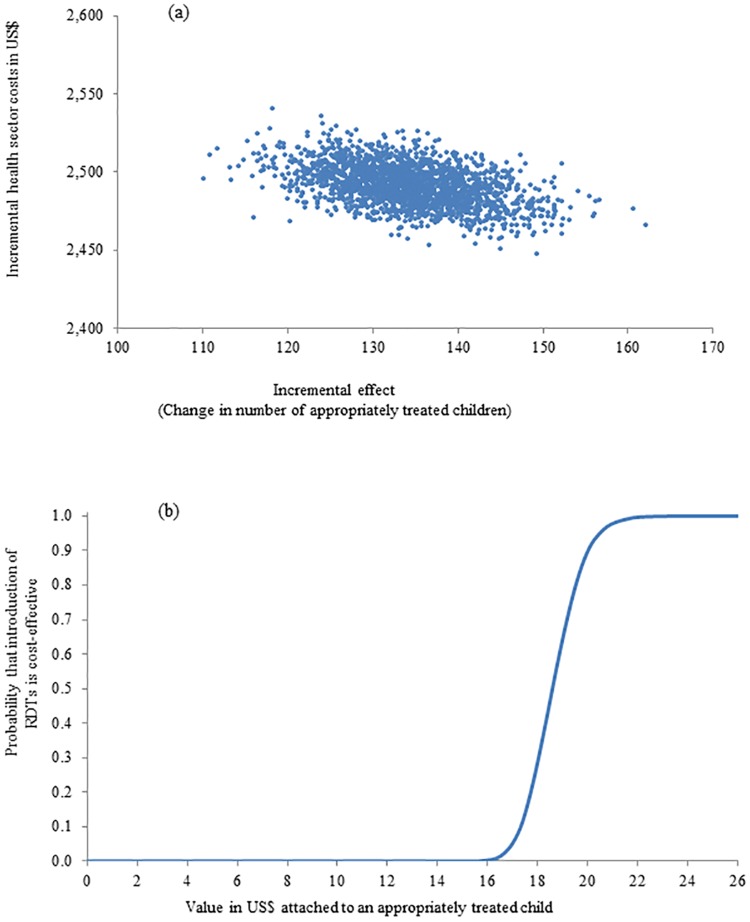
Probabilistic sensitivity analysis (health sector perspective): (a) scatter plot of incremental health sector cost in US$ and incremental number of appropriately treated fever episodes resulting from replacing clinical diagnosis of malaria by rapid diagnostic test, 2011 (US$1 = GHS1.51) and (b) cost-effectiveness acceptability curve.

**Fig 4:**
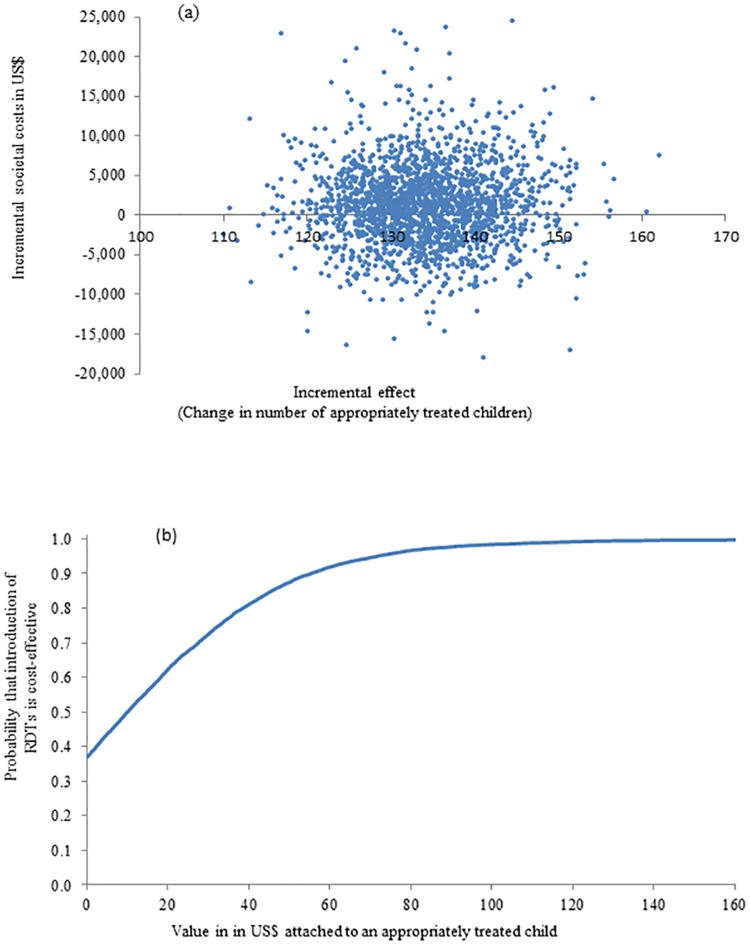
Probabilistic sensitivity analysis (societal perspective): (a) scatter plot of incremental societal cost in US$ and incremental number of appropriately treated fever episodes resulting from replacing clinical diagnosis of malaria by rapid diagnostic test, 2011 (US$1 = GHS1.51) and (b) cost-effectiveness acceptability curve.

The results of the probabilistic sensitivity analyses are presented in Figs 3 and 4. From a health sector perspective, all pairs of incremental health sector cost and effects were situated in the north-eastern quadrant of the cost-effectiveness plane meaning that the change in number of appropriately treated children was always positive and incremental health sector cost always positive if mRDTs were introduced (Fig 3, Panel a). The cost-effectiveness acceptability curve (CEAC) from a health sector perspective (Fig 3, Panel b) displayed the probability that the introduction of mRDTs in public health centres was cost-effective given different levels of a health policy maker’s hypothetical willingness to pay (WTP) for an appropriately treated fever episode. If the health policy maker was willing to pay at least US$20 per appropriately treated child, the probability of mRDT introduction being a cost-effective intervention was 90% with this probability becoming 98% and 100% if WTP was US$21 and US$22. From a societal perspective, the cost-effectiveness scatter plot exhibited considerably more variation (uncertainty) in costs compared to the health sector perspective as seen by much larger spread and spanning both positive and negative incremental societal costs (Fig 4, Panel a). In the south-eastern quadrant where the mRDT intervention had a higher number of appropriately treated fever episodes and lower costs than the clinical judgement intervention, the introduction of mRDTs would therefore be the dominant intervention from a cost-effectiveness viewpoint. According to the CEAC from a societal perspective (Fig 4, Panel b), if the WTP of a policy maker was US$20, the probability that mRDT introduction was cost-effective would be 62% increasing to 72% and 81% if the WTP was US$30 and US$40 respectively.

## Discussion

This cost-effectiveness analysis used a decision model populated with primary data from a cluster-randomised trial [23] supplemented with cost data collected in Ghana. The aim was to evaluate the cost-effectiveness of introducing mRDTs in lower level public health centres where malaria diagnosis was typically presumptive.

The results of this research provided support for the introduction of mRDTs in public health centres instead of presumptive malaria diagnosis. Test-based management of suspected malaria led to an increase in appropriately treated children by 13.4 percentage points compared to clinical judgement. This improvement was obtained at low cost to the health sector of US$18.6 per additional appropriately treated child and even lower societal cost of US$11.0 per appropriately treated child if costs borne by households were included. In addition, the extra cost per child with fever managed with mRDT rather than presumptively was only US$2.49 from a health sector perspective and US$1.47 from a societal perspective. A study conducted in public health centres in another area of Ghana found that the incremental cost per appropriately treated fever patient was US$10.5 from the health sector perspective and US$8.5 from a societal perspective [16]. In Afghanistan with extremely low malaria transmission, incremental cost per appropriately treated fever patient was estimated to be US$2.5 and US$4.5 from the health sector and societal perspectives respectively [40]. A possible reason for the higher ICER levels in Kintampo compared to the other studies was high malaria positivity rate among children with fever. High malaria transmission will tend to limit the potential of mRDTs to improve appropriate malaria treatment compared to clinical judgement and therefore increase the ICER level. While the ICER levels in Kintampo therefore appear to be at the high end compared to other studies which were conducted in areas with lower malaria transmission, it will ultimately be up to health policy makers in Ghana to decide if the level of cost per extra appropriately treated fever child found in this study is good use of government and societal resources compared to competing beneficial health interventions.

While ICERs were robust to variations in most of the model parameters, the one-way sensitivity analyses also identified aspects worth considering before deciding to introduce mRDTs. First, a high adherence to mRDT results was essential to ensure the relative cost-effectiveness of mRDTs over clinical judgement and an almost complete adherence to negative mRDTs was found in this study due to the trial setting. A decline in adherence to negative mRDTs to 50% as experienced in another study in Ghana [16] would lead to more than doubling the ICERs of the present study. This suggests that efforts to maintain high adherence among health personnel such as regular or thorough training could be valuable use of resources as found in a study in Cameroon [41]. Second, it was important that the mRDT chosen was of high accuracy to ensure appropriate treatment and reduced ACT cost. If the mRDT specificity had been 90% rather than the actual 47% of this study, then the introduction of mRDTs would have decreased ACT cost by 34% instead of the actual 9% observed and improved the cost-effectiveness of mRDTs. Third, ICERs were sensitive to the level of malaria positivity rate among children visiting health centres. The lower the positivity rate, the more cost-effective mRDTs were found to be. At positivity rates considerably higher than the 50% found in the present study such as 80% or 90%, the introduction of mRDTs would become increasingly more questionable from a cost-effectiveness viewpoint. However, with the increased efforts for elimination of malaria, the positivity of malaria among fever cases is likely to decrease and thus decrease the ICER.

Household cost borne by families to children in the mRDT arm was lower than in the clinical judgement arm explaining why the ICER was lower from a societal perspective compared to a health sector viewpoint (US$11.0 versus US$18.6). However, this research also found large variation in household costs in both arms leading as a consequence to a large variation in societal cost as displayed in Fig 4 (Panel a) and the wide confidence interval for incremental societal cost. Such high variation in household cost led to higher uncertainty for the health policy makers in the decision to introduce mRDTs or not from a societal viewpoint: If the value per appropriately treated child was agreed to be US$20, then there was a 90% probability that the introduction of mRDTs was a cost-effective intervention from a health sector perspective (Fig 3, Panel b) while there was only a 62% probability that the introduction of mRDTs was cost-effective from a societal perspective where the impact on households were also taken into consideration (Fig 4, Panel b). There might be several possible explanations for this variation in household cost. First, there could genuinely be a large variation in responses to children’s illnesses in individual households with respect to additional treatment-seeking, purchasing food believed to help the child to become better and taking time to care for an ill child. Recall bias on out-of-pocket expenditure and time utilised should be minimal as interviews were conducted at the time of health centre visit and subsequently in the homes of the children with fever 3 and 7 days later. Respondents therefore only had to remember a few days back in time. Second, there were other possible factors contributing to the variation in household cost. For instance, respondents might find it difficult to give an exact estimate on ‘the number of days unable to perform normal activities’ since caring for the child might not take up the whole day enabling family members to perform some of their normal activities. This question may therefore be open to different interpretations by respondents. An analysis of the replies to this particular question suggested that some respondents equated the time unable to do normal activities as the full illness period of the child while other respondents gave a reply corresponding to the days where the child was severely ill and in need of much care. Further, household cost interviews were conducted across the year thereby incorporating both high and low malaria transmission periods. The response to fever illnesses and thereby household cost could be different in the high and the low season introducing variation in (average) household cost.

This study estimated an ICER from a societal perspective that was close to half the level of the ICER from the health sector perspective. This finding highlighted the importance of taking the wider societal perspective in a cost-effectiveness analysis incorporating the cost borne by other sectors of the economy than the health sector, in particular households affected by an intervention. The introduction of mRDTs increased the cost to the health care sector despite lower cost from reduced ACT consumption compared to clinical judgement of malaria. At the same time, this study suggested that the utilisation of mRDTs at health centres lessened the burden on households thereby leading to a reduction of societal cost. Families in the mRDT arm experienced lower out-of-pocket expenditure due to a reported lower tendency to seek extra care after a health centre visit and fewer days occupied by taking care of ill children. An added advantage to families in the mRDT arm was a lower risk of exposure to health providers such as drug shops where parasitological diagnosis is rarely available and less effective antimalarials are often sold [42]. As mentioned earlier, high variation in household cost was experienced in the sample of 2006 household interviews which constituted only 18% of the total episodes incorporated in the trial to measure the effect. This suggested investing in a higher sample size for investigating household cost in future studies.

## Limitations

The research project provided free membership of the National Health Insurance Scheme (NHIS) to all households with children participating in the trial. This effectively reduced the point-of-care costs for the households when seeking care (at accredited health providers) especially among households that had not planned to purchase insurance membership during the trial period. This might have led to a different treatment-seeking behaviour and lower reported household costs compared to a non-trial situation. However, reduced point-of-care costs may be more common in the future as membership of the NHIS is compulsory and the share of households being scheme members will likely increase and eventually cover the majority of the population.

The cost of hospital inpatient care was not included in the analysis even though the unit cost of an inpatient stay would be much higher than a health centre outpatient visit. Trial data suggested that the number of admissions for inpatient care was low and at similar levels in the two study arms. The measurement and inclusion of hospital inpatient costs was therefore unlikely to change the conclusions made.

## Conclusions

The introduction of mRDTs into public health centres is likely to be considered a cost-effective intervention in this high malaria transmission setting: a test-based diagnosis led to a higher number of appropriately treated fever episodes compared to clinical judgement at relatively low health sector cost of US$18.6 per additional fever episode appropriately treated and even lower societal cost of US$11.0. Whether this cost is sufficiently low compared to the cost of other competing, beneficial health sector interventions will ultimately be the decision of policy makers in Ghana, and other countries with similar levels of malaria transmission and health system. The relative cost-effectiveness of a test-based approach could be improved further if an mRDT is identified with higher specificity without compromising its sensitivity. The test-based approach also appeared to be a benefit to families as caregivers reported lower levels of out-of-pocket expenditure and less time utilised for caring for ill children if they had visited a health centre offering mRDT diagnosis compared to households who attended health centres with clinical management of malaria.
