# Correction: Cost-Effectiveness Analysis of Test-Based versus Presumptive Treatment of Uncomplicated Malaria in Children under Five Years in an Area of High Transmission in Central Ghana

**DOI:** 10.1371/journal.pone.0170848

**Published:** 2017-01-20

**Authors:** The PLOS ONE Staff

In Table 3, there are several missing data points. Please see the corrected Table 3 here. The publisher apologizes for the errors.

**Table 3:** Sensitivity to selected parameters of the incremental cost-effectiveness ratio (ICER) of replacing clinical diagnosis of malaria by rapid diagnostic tests in public health centres in Kintampo District, Ghana, July 2010-June 2012 (US$1 = GHS1.51).

	------- ICER in US$ -------			------- ICER in US$ -------		
Parameter ^&^	Health sector	Societal	Parameter ^&^	Health sector		Societal
*Malaria prevalence among*			*Health centre cost per*			
*children with fever (50%)*			*visit excl drugs and mRDTs*			
20%	13.0	7.4	40% lower	13.9		6.3
30%	14.5	8.3	20% lower	16.2		8.7
40%	16.2	9.5	20% higher	20.9		13.3
60%	21.1	12.6	40% higher	23.3		15.7
70%	24.6	14.9	*Training and supervision*			
80%	29.3	17.9	*cost per episode*			
*mRDT sensitivity (95%)*			40% lower	18.1		10.5
80%	36.3	20.7	20% lower	18.3		10.8
90%	21.9	12.8	20% higher	18.8		11.2
100%	16.0	9.6	40% higher	19.1		11.5
*mRDT specificity (47%)*			*Opportunity cost*			
60%	12.3	7.1	*per day (US$1*.*14)*			
75%	8.6	4.8	US$0.00	18.6		14.6
90%	6.5	3.4	US$0.50	18.6		13.0
100%	5.5	2.8	US$1.00	18.6		11.4
*Sensitivity / specificity*			US$1.50	18.6		9.8
100% / 40%	20.7	12.5	US$2.00	18.6		8.2
90% / 50%	19.4	11.3	US$2.50	18.6		6.7
80% / 60%	18.2	10.0	*Probability of additional*			
*Adherence to negative*			*treatment-seeking in*			
*mRDT result (96%)*			*test-based arm (11%)*			
60%	46.5	28.7	5%	18.6		10.0
70%	33.5	20.4	20%	18.6		12.4
80%	25.9	15.6	30%	18.6		14.0
90%	21.0	12.5	*Probability of additional*			
100%	17.6	10.3	*treatment-seeking in*			
*mRDT price (US$0*.*82)*			*clinical judgement arm (19%)*			
US$0.20	13.6	6.0	5%	18.6		13.1
US$0.40	15.1	7.5	20%	18.6		10.9
US$0.60	16.6	9.0	30%	18.6		9.4
*ACT price (US$1*.*32)*			*Various household costs*			
US$0.80	18.9	11.3	*per episode*			
US$1.00	18.8	11.2	Special food equal by arm		18.6	13.6
US$1.20	18.7	11.1	Days lost equal by arm	18.6		14.3

^&^ Central parameter values are shown in parentheses.
